# Contrasting Effects of Short-Term Mediterranean and Vegan Diets on Microvascular Function and Cholesterol in Younger Adults: A Comparative Pilot Study

**DOI:** 10.3390/nu10121897

**Published:** 2018-12-03

**Authors:** David Rogerson, Diana Maçãs, Marianne Milner, Yingshan Liu, Markos Klonizakis

**Affiliations:** 1 Academy of Sport and Physical Activity, Sheffield Hallam University, Sheffield S10 2BP, UK; d.rogerson@shu.ac.uk; 2 The University of Sheffield, Sheffield S10 2RX, UK; dfjmacas1@sheffield.ac.uk (D.M.); marrianebmilner@gmail.com (M.M.); liuyingshan_sylvia@hotmail.com (Y.L.); 3 Centre for Sport and Exercise Sciences, Sheffield Hallam University, Sheffield S10 2BP, UK

**Keywords:** vegan diet, mediterranean diet, microvascular function, laser doppler flowmetry, cholesterol, cardiovascular disease

## Abstract

The Mediterranean diet has been shown to improve cardiovascular health. Vegan diets have demonstrated similar benefits, albeit in fewer studies. In a comparative pilot study, we compared the effects of a short-term Mediterranean Diet (MD) and Vegan Diet (VD) on microvascular function and cholesterol levels in a healthy population. Twenty-four young (aged 18 to 35 years) healthy volunteers followed a four-week intervention (MD = 12; VD = 12) ad libitum. Pre and post-intervention anthropometrics, microvascular function (assessed via LDF and expressed as raw CVC and %CVC MAX), dietary-analysis data (Calories, Protein, Carbohydrates, Total Fat, Saturated Fat, Fibre), Mean Arterial Pressure (MAP), Blood Pressure, Total Cholesterol (TC), High Density Lipoprotein (HDL-C) and TC:HDL-C were compared. MD participants reduced Total Fat intake (*p* = 0.05). Saturated Fat decreased (MD: *p* = < 0.001; VD: *p* = 0.004) and Fibre increased (MD: *p* = 0.02; VD: *p* = < 0.001) in both groups. Dietary changes reflected improvements in plateau raw CVC in the MD group (*p* = 0.005), and a reduction in TC (*p* = 0.045) and weight loss (*p* = 0.047) in the VD group. The MD led to improvements in microvascular function; the VD led to reduced TC and weight loss. Although both diets might offer CVD risk-reduction benefits, evidence for the MD appeared to be stronger due to changes in vasodilatory ability and NO bioavailability.

## 1. Introduction

Cardiovascular disease (CVD) is a leading cause of death responsible for >30% of mortalities worldwide [1]. Sedentariness, alcohol consumption, poor diet, and smoking are common lifestyle risk factors linked to CVD [1,2,3]. These factors contribute to high blood pressure, elevated lipids, and obesity—conditions that appear to be receptive to lifestyle modifications such as improved diet and increased physical activity. Increasing fruit and vegetable consumption is a simple lifestyle adjustment that can reduce the risk of CVD if prior intake is insufficient [4]. However, few appear to achieve recommended intakes [5], necessitating strategies to foster this important behaviour change. The Mediterranean Diet (MD) is a diet that promotes fruit and vegetable consumption (in addition to oily fish, olive oil, red wine, lean meats, nuts, and low-fat dairy products) that might have cardio-protective benefits [6,7,8,9,10]. The MD has been shown to improve cholesterol [8]; reduce overall mortality [9]; and mortality from CVD specifically [10]. These benefits have been attributed to increases in micronutrients, reductions in saturated fat and increases in olive oil fostering improved cardiovascular health [7,8,9,10,11,12]. 

Vegetarians opt to consume a meat-free diet, which, through its emphasis on plant-based foods, might also be cardio-protective [12]. Vegans follow a stricter vegetarian diet and abstain from eggs, dairy, and honey in addition to flesh. Early data indicates that a Vegan Diet (VD) is associated with lower BMI and reduced insulin resistance [12,13]. Literature has also shown that a VD reduces CVD risk [14,15], and vegans possess lower total cholesterol, low-density lipoprotein, and high-density lipoprotein (HDL) than non-vegans [16,17]. However, despite possible benefits, vegan diets appear to be under-investigated empirically, and comparisons between VDs and other novel diets, such as the MD, have yet to be undertaken it appears. Research into the effects of short-term interventions is also uncommon; however, encouraging data from our lab has shown that improvements in microvascular health markers can be observed after only two weeks of dietary modification [18]. 

Poor microvascular functioning is linked to increased CVD risk [18,19,20], specifically if endothelial dysfunction presents with other systemic risk factors such as high cholesterol [21]. Assessing microvascular function in conjunction with such risk factors is a useful metric for assessing cardiovascular health and evaluating CVD risk [21]. We therefore compared the effects of a four-week MD vs. VD intervention on microvascular function and cholesterol levels in young sedentary individuals to investigate potential cardiovascular-health effects of two short-term dietary interventions in a comparative pilot investigation. We hypothesised that both diets would lead to improvements in CVD markers but that effects might differ on the basis of dietary composition (via the inclusion vs. exclusion of animal products, etc.).

## 2. Materials and Methods

### 2.1. Ethical Approval

Ethical approval for this study was granted by Sheffield Hallam University’s Health and Wellbeing Research Ethics Committee (ethical approval code HWB-2016-17-S&E-43) and was conducted in accordance with the revised Declaration of Helsinki.

### 2.2. Participants

Twenty-four individuals (MD = 12; VD = 12) provided written informed consent to participate in this research. Participants were recruited to each treatment arm (MD vs. VD) preintervention, to foster adherence and reduce attrition. Adherence and completion of dietary interventions can be an issue for some studies, particularly if a treatment requires participants to make large changes to their habitual eating habits, such as a vegan diet. We therefore recruited individuals motivated to participate in each arm pragmatically, which we identified through advertisement throughout the local region. To achieve our low attrition rate, we used our ‘six pillars of adherence’ strategy (based upon ‘social support’, ‘education’, ‘reachability’, ‘small groups intervention implementation’, ‘reminders’, and ‘simplicity’), which we have used previously with excellent results in lifestyle interventions (i.e., over 90% of retention and 79% of completion) [22,23].

All participants were required to be aged between 18 and 35 years and be healthy and sedentary. We accounted for and excluded for all known risk factors that affect microcirculation, e.g., smoking, high blood pressure, unhealthy lipid profile, diabetes, and known cardiovascular or microcirculatory diseases during recruitment. Sedentariness was assessed using the International Physical Activity Questionnaire (IPAQ) and defined as undertaking ≤60 minutes of structured/planned physical activity per week. Health status was assessed via a pre-test medical questionnaire. Habitual diet was evaluated by two 14-item questionnaires (one for the MD and VD) that measured food frequency. The 14-item MD questionnaire has been described elsewhere [24]. The 14-item VD questionnaire was developed for this study specifically and reflected the MD questionnaire in its format and calculation. A score of ≤ 6 for either questionnaire indicated low habitual adherence and was a criterion for inclusion. Female participants were studied specifically during days 1–7 of their menstrual cycle, to minimise the influence of cyclical changes in female sex hormones on our findings [25].

### 2.3. Dietary Intervention

Participants were instructed to follow the diets for four weeks, with advice and guidance provided via email, face-to-face and/or telephone consultation throughout the intervention period. A three-day food diary was completed to assess changes in nutritional data pre and post intervention. Data was inputted into and analysed using Nutritics™ (Nutritics Ltd, Co. Dublin, Ireland), dietary analysis software (Education, Nutritics, Dublin, Ireland) and its proprietary databank (Nutritics Ltd product version 1.7, Dublin, Ireland). Participants were encouraged to follow the diets (Table 1) without restricting energy. The MD was based on the intervention developed by Estruch et al. [26]; the VD was developed based on the recommendations of Phillips [27]. Both groups were provided with resources, including guidance documents, shopping lists, and recipes prior to commencing their intervention. Food items were provided to assist adherence. 

### 2.4. Anthropometric Assessments

Participants’ measurements were assessed pre and post intervention using procedures highlighted elsewhere [28]. Body mass (kg) was measured using balance beam scales (Adam GFK 150H, Adam Equipment Co. Ltd., Milton Keynes; UK) to the nearest 0.05 kg. Stature (m) was measured using a wall-mounted stadiometer (Harpenden, UK) to the nearest 0.01 cm. Body mass index (BMI) was calculated as: $BMI=\frac{Body\;Mass\;\left(kg\right)}{Stature\;\left(m^{2}\right)}$.

### 2.5. Microvascular Assessments

Microvascular blood flow was measured via Laser Doppler Flowmetry (Periflux system 5000, Perimed 122 AB, Järfälla, Sweden) using procedures previously described by our lab [18], and by Tew et al. [29]. Readers are advised to consult these articles for detailed descriptions of the LDF procedure. Microvascular blood flow data were expressed as cutaneous vascular conductance (CVC) at baseline, initial peak, plateau, and maximum vasodilation stages. These values were presented as raw CVC and CVC normalised to maximum: $\%CVC_{max}=\frac{CVC}{CVC_{max}}\times 100$.

### 2.6. Cholesterol Assessment

Finger-prick blood-capillary samples were obtained pre and post intervention using a Cholestec monitoring device (Cholestec LDX systems) in a fasted state, to determine values for Total Cholesterol (TC), High Density Lipoprotein (HDL-C), and TC:HDL-C, using previously-documented procedures [28].

### 2.7. Statistical Analysis

Independent *t*-tests were performed on physical characteristics (Stature, Body Mass, BMI, Systolic, and Diastolic blood pressure), nutritional data (Calories, Carbohydrates, Protein, Total Fat, Monounsaturated Fat, Polyunsaturated Fat, Omega-3 fatty acids, Omega-6 fatty acids, Saturated Fat and Fibre); micronutrients and food groups (Dairy, Meat and Poultry, Vegetables, etc.), Mean Arterial Pressure (MAP), Raw CVC and %CVC MAX (Baseline, Initial Peak, and Plateau) and Cholesterol (TC, HDL-C, and TC:HDL-C) data pre and post intervention. Dependent *t*-tests were performed to determine pre-to-post changes of following the MD or VD. The difference in pre and post intervention scores (∆) was calculated (test 2−test 1) for each dependent variable. Independent *t*-tests were then performed on this data. To satisfy normality, log transformations were undertaken for several variables (Carbohydrate, Saturated Fat, ∆Calories, BMI, TC:HDL-C, Baseline %CVC MAX, Initial Peak %CVC MAX ∆Raw CVC Plateau, ∆Baseline %CVC MAX and ∆%CVC MAX Plateau). Where homogeneity of variance or normality could not be assumed (∆ Saturated Fat, Food Groups, ∆Sodium, ∆Phosphorous, ∆Magnesium, ∆Iron, ∆Vitamin K, ∆Thiamine, ∆Riboflavin, ∆Vitamin C, ∆Vitamin B12, Raw CVC Initial Peak and Plateau (pre intervention), Initial Peak %CVC MAX ((log transformed) and ∆Raw CVC)), the Mann–Whitney U test was undertaken to assess between-groups comparisons; the Wilcoxon signed-rank test was performed for within-groups analyses. Cohen’s *d* (standardised mean differences) was calculated as ($\frac{{\bar{X}}_{1}-{\bar{X}}_{2}}{SD_{Pooled}}$) for each dependent variable from the raw data, and effect sizes of 0.2, 0.5 and 0.8 were interpreted as being small, medium or large, reflecting Cohen’s criteria [30]. SPSS version 24 (SPSS Inc., Chicago, IL, USA) was used for the analyses. Data are presented as mean ± SD. The alpha level was set to *p* = ≤ 0.05 a priori.

## 3. Results

### 3.1. Participants

Twelve (MD) and eleven (VD) participants completed from the twenty four recruited initially, equating to a 100% (MD) and 92% (VD) completion rate. 

### 3.2. Body Mass, BMI, Blood Pressure, and MAP

Pre- and post-intervention values for body mass and BMI were similar for both groups. The VD group experienced weight loss post intervention (Body Mass Visit 1 = 73.2 ± 18.6, Body Mass Visit 2 = 72.1 ± 17.6; *p* = 0.047; *d* = −0.08), and ∆ Body Mass (MD = 0.17 ± 1.06, VD = −1.05 ± 1.53; *p* = 0.04; *d* = 1.3) and ∆ BMI (MD = 0.06 ± 0.36, VD = −0.36 ± 0.54, *p* = 0.04; *d* = 1.3) scores indicated that the VD and MD groups experienced different Body Mass and BMI changes as a result of their diets (Table 2).

Baseline Systolic (MD = 107.88 ± 8.83, VD = 119.00 ± 9.48; *p* = 0.01; *d* = −1.7) and Diastolic blood pressure (MD = 68.20 ± 7.28, VD = 76.4 ± 10.8; *p* = 0.03; *d* = −1.3) was lower in the MD group. Post intervention, both Systolic (MD = 108.82 ± 7.82, VD = 117.55 ± 13.00; *p* = 0.05; *d* = −1.2) and Diastolic blood pressure (MD = 68.12 ± 6.29, VD = 77.09 ± 13.00; *p* = 0.05; *d* = −1.2) remained lower for the MD group. Neither diet affected blood pressure or MAP (Table 2).

### 3.3. Total Cholesterol, HDL-C, and TC: HDL-C

#### 3.3.1. Total Cholesterol

Baseline (MD = 4.20 ± 0.67, VD = 3.88 ± 0.56, *p* = 0.02; *d* = 0.73) and post- intervention (MD = 4.15 ± 0.75, VD = 3.52; *p* = 0.05; *d* = 1.22) TC values differed between groups, with MD participants exhibiting larger values pre intervention (Table 2).

VD participants experienced a noteworthy reduction in TC post intervention (Visit 1 = 3.88 ± 0.56, Visit 2 = 3.52 ± 0.71; *p* = 0.045; *d* = −0.8) however ∆ TC did not differ between groups (Table 2).

#### 3.3.2. HDL-C

HDL-C levels were similar pre and post-intervention for both groups, and neither group experienced notable changes in HDL-C (Table 2).

#### 3.3.3. TC: HDL-C

TC: HDL-C values were similar for both group pre and post intervention, and TC: HDL-C did not appear to change in either group (Table 2).

### 3.4. Nutritional Data

#### 3.4.1. Food Groups

Apart from Olive Oil (MD = 0 ± 0, VD = 7 ± 6.3; *p* = 0.028, *d* = 0.66) there were no differences between the groups for Food Group consumption at the pre-intervention stage. Post intervention, Meat and Poultry (MD = 132.7 ± 176.8, VD = 0 ± 0; *p* = 0.043; *d* = 1.43) and Fish (MD = 41.9 ± 36.9; VD = 0 ± 0; *p* = 0.013; *d* = 2.13) consumption was greater in the MD group; ∆ Other Oils and Fats (MD = −13.4. ± 20.4, VD = −4.28 ± 9.7; *p* = 0.010, *d* = −0.56) indicated that both groups consumed fewer Oils and Fats by undertaking their intervention, but that this effect was greater in the MD group. In contrast, VD participants consumed more Fruit (MD = 187.8 ± 123.9, VD = 401.5 ± 352.5; *p* = 0.043; *d* = 0.92), which was a greater change in consumption habits for this group (∆ Fruit MD = 170.2 ± 143.5, ∆ Fruit VD = 280.1 ± 357.9; *p* = 0.022; *d* = 0.48) (Table 3).

Between visits, the MD group increased their consumption of Vegetables (Visit 1 = 165.2 ± 142.5, Visit 2 = 263.3 ± 83.6; *p* = 0.038, *d* = 0.9) and Olive Oil (Visit 1 = 0 ± 0, Visit 2 = 14.7 ± 6.9; *p* = 0.018, *d* = 4.2), and reduced their intake of Other Oils and Fats (Visit 1 = 16.5 ± 16.2, Visit 2 = 3.1 ± 6.9; *p* = 0.08 *d* = −1.2) and Alcohol (Visit 1 = 5.3 ± 9.2, Visit 2 = 1.9 ± 5.7; *p* = 0.012, *d* = 0.6). The VD group eliminated all consumption of Meat and Poultry (Visit 1 = 173.3 ± 264.4, Visit 2 = 0 ± 0; *p* = 0.028, *d* = −1.31) and Eggs (Visit 1 = 57.5 ± 28.4, Visit 2 = 0 ± 0; *p* = 0.42, *d* = −4.05); However Fish consumption did not reach statistical significance, despite being eliminated from the diet. Vegetable consumption increased (Visit 1 = 132.1 ± 11.6, Visit 2 = 279.1 ± 165; *p* = 0.012, *d* = 1.06), along with Fruit (Visit 1 = 121.4 ± 114.1, Visit 2 = 401.5 ± 352.5); *p* = 0.028, *d* = 1.20) and Olive Oil (Visit 1 = 7 ± 6.3, Visit 2 = 8.2 ± 6.2; *p* = 0.013, *d* = 0.19); however Refined Cereal consumption decreased (Visit 1 = 36 ± 68, Visit 2 = 15 ± 18.9); *p* = 0.038, *d* = −0.48).

#### 3.4.2. Energy, Macronutrients, and Fibre

Baseline data for Calories, Carbohydrates and Protein, Total Fat, Monunsaturated Fat, Poyunsaturated Fat, Omega-3 Fatty Acids, Omega-6 Fatty Acids, Saturated Fat, and Fibre values did not differ between the groups, suggesting parity for pre-intervention nutrition. Post intervention, differences in Protein (MD = 82.3 ± 31.3, VD = 57.2 ± 18.6; *p* = 0.04; *d* = 1.2) and Fibre (MD = 26.94 ± 6.34, VD = 37.7 ± 11.9; *p* = 0.02; *d* = −1.6) emerged (Table 4). The VD group ate less protein by adopting the VD intervention compared to pre-intervention habits (Visit 1 = 82.3 ± 31.3, Visit 2 = 57.2 ± 18.6, *p* = 0.05; *d* = −1.31); however, between-group comparisons for ∆ Protein did not reach statistical significance.

The MD group reduced Total Fat by following the diet (Visit 1 = 88.2 ± 32.8, Visit 2 = 73.8 ± 28.8, *p* = 0.05; *d* = −0.66); ∆ Total Fat did not differ between groups however. Both groups reduced Saturated Fat (Log10 Saturated Fat MD: Visit 1 = 1.45 ± 0.28, Visit 2: 1.34 ± 0.4, *p* = 0.001; *d* = −1.2; Saturated Fat VD: Visit 1 = 38.2 ± 22.1, Visit 2 = 14.5 ± 5.6, *p* = 0.004; *d* = −2.1), and ∆ Saturated Fat (MD = −10.72 ± 6.39, VD = −23.62 ± 19.45; *p* = 0.07; *d* = 1.26) differed between groups. While both interventions led to an increase in Fibre (MD: Visit 1 = 20.1 ± 5.8, Visit 2 = 26.9 ± 6.7, *p* = 0.02; *d* = 1.38; VD: Visit 1 = 20.5 ± 6.3, Visit 2 = 37.7 ± 11.9, *p* < 0.001; *d* = 2.56), ∆ Fibre (MD = 6.17 ± 5.27, VD = 17.19 ± 10.83; *p* < 0.001; *d* = −1.83) suggested that the VD group experienced the greatest change. No other findings were observed. Data are presented in Table 4.

#### 3.4.3. Micronutrients

Magnesium intakes differed between the groups at the pre-intervention stage (MD = 423 ± 135, VD = 283.9 ± 104, *p* = 0.02; *d* = 1.1) however no other micronutrient intake differed at this point. Post-intervention, differences emerged for Sodium (MD = 2146 ± (785), VD = 1161 ± 852, *p* = 0.018; *d* = 1.10), ∆ Sodium (MD = 577 ± (914), VD = −931 (586), *p* = ≥ 0.001; *d* = 1.95), Chloride (MD = 3612 ± (1343), VD = 1512 ± 478, *p* ≤ 0.001; *d* = 2.25), ∆ Chloride (MD = 919 ± 713, VD = −1455 ± 1267, *p* ≤ 0.001; *d* = 2.16), ∆Calcium (MD = 89 ± 211, VD = −318 ± 455, *p* = 0.025; *d* = −1.53), ∆Magnesium (MD = −11 ± 99, VD = 91 ± 98, *p* = 0037; d = −2.96), ∆Zinc (MD = 0.8 ± 2, VD = −2.2 ± 3.5, *p* = 0.031; d = −1.29), ∆ Copper (MD = 0.3 ± 0, VD = 0.8 ± 0.5, *p* = 0.016; *d* = −1.58), Manganese (MD = 3.8 ± 1, VD = 5.8 ± 1.9, *p* = 0.007; *d* = 0.82), Selenium (MD = 65.7 ± 34, VD = 38.1 ± 18.4, *p* = 0.038; d = −0.60), Iodine (MD = 131 ± 49, VD = 19.05 ± 6.9, *p* ≤ 0.001; *d* = −1.62), Riboflavin (MD = 1.7 ± 1, VD = 1.1 ± 0.5, *p* = 0.014; *d* = 0.50), Niacin (MD = 47.9 ± 28, VD = 23 ± 11, *p* = 0.03; *d* = 1.34), Pantothenic Acid (MD = 6.3 ± 3, VD = 3.5 ± 1, *p* = 0.02; *d* = −0.73), Vitamin B6 (MD = 2.3 ± 1, VD = 1.4 ± 0.4, *p* = 0.025; *d* = −0.60), and Vitamin B12 (MD = 5 ± 2; VD = 0.9 ± 1, *p* ≤ 0.001; *d* = −1.33), suggesting that the adoption of the interventions altered participants’ micronutrient consumption habits. Data are presented in Table 5.

The MD group consumed more Chloride (Visit 1 = 2693 ± 1092, Visit 2 = 3612 ± 1343, *p* = 0.005; *d* = 0.75), Iron (Visit 1 = 12.1 ± 3, Visit 2 = 13.6 ± 3, *p* = 0.04; *d* = 0.49), Manganese (Visit 1 = 3.2 ± 1, Visit 2 = 3.8 ± 1, *p* = 0.007; *p* = 1.04), Vitamin A (Visit 1 = 627 ± 438, Visit 2 = 1054 ± 562, *p* = 0.015; *d* = 0.85), Thiamine (Visit 1 = 1.5 ± 0, Visit 2 = 2.2 ± 1, *p* = 0.015; *d* = 1.02), and Biotin (Visit 1 = 34.1 ± 14, Visit 2 = 36 ± 6, *p* = 0.038, *d* = 0.17 (Table 4) by following their intervention. 

VD participants consumed less Sodium (Visit 1 = 2092 ± 1080, Visit 2 = 1161 ± 852, *p* = 0.001; *d* = −0.96), Chloride (Visit 1 = 2967 ± 1394, Visit 2 = 1512 ± 478, *p* = 0.005; *d* = −1.6), Phoshporous (Visit 1 = 1367.6 ± 491, Visit 2 = 1020 ± 269, *p* = 0.0364; *d* = −0.95), Iodine (Visit 1 = 169.3 ± 190.6, Visit 2 = 19.05 ± 6.9, *p* = 0.03; *d* = −1.52), Riboflavin (Visit 1 = 1.7 ± 0.9, Visit 2 = 1.1 ± 0.5, *p* = 0.016, *d* = −2.9), and Vitamin B12 (Visit 1 = 4.9 ± 3, Visit 2 = 0.9 ± 1, *p* ≤ 0.001; *d* = −2.04) from following the Vegan Diet. However, consumption of Magnesium (Visit 1 = 283.9 ± 104, Visit 2 = 375 ± 139, *p* = 0.016; *d* = 0.75), Iron (Visit 1 = 9.7 ± 3.4, Visit 2 = 13.5 ± 5, *p* = 0.03; *d* = 0.90), Copper (Visit 1 = 1.1 ± 0.4, Visit 2 = 1.9 ± 0.8, *p* ≤ 0.00; *d* = 1.46), and Manganese (Visit 1 = 3.6 ± 1.4, Visit 2 = 5.8 ± 1.9, *p* = 0.004; *d* = 1.379) increased (Table 5). 

### 3.5. Cutaneous Vascular Conductance

#### 3.5.1. Baseline

##### Raw CVC

Raw CVC data did not differ between groups at baseline nor post intervention (Table 6).

##### %CVC MAX

Baseline %CVC MAX values were greater in the VD group but not statistically significant. Post intervention, both groups experienced a marginal but non-significant decrease in %CVC MAX (Table 6).

#### 3.5.2. Initial Peak

##### Raw CVC

Initial peak Raw CVC values at baseline were similar for both groups. Post intervention, despite differences in mean data, between-group differences did not reach statistical significance. ∆ Raw CVC differences were evident however (MD = 0.90 ± 0.8, VD = 0.09 ± 0.9; *p* = 0.04; *d* = 1.32) and suggested that the MD led to greater increases from pre-intervention levels (Table 6).

##### %CVC MAX

The VD group exhibited a lower but non-significant %CVC MAX at baseline (Table 4). Post intervention data did not reach statistical significance. ∆ %CVC MAX did not differ between groups (Table 6).

#### 3.5.3. Plateau

##### Raw CVC

Plateau Raw CVC values were similar for both groups pre intervention. Post-intervention, raw CVC for the MD group increased significantly (Visit 1 = 2.59 ± 0.67, Visit 2 = 3.32 ± 0.8; *p* = 0.005; *d* = 1.02). There was no significant change in raw CVC between visits for the VD group. Comparisons between the groups for ∆ Raw CVC data did not reach statistical significance (Table 6).

##### %CVC MAX

Baseline and post-intervention plateau %CVC MAX demonstrated no between-group differences. While both groups experienced a marginal decline in %CVC MAX, neither group experienced statistically-significant changes. No differences in ∆ %CVC MAX were observed between groups (Table 6).

## 4. Discussion

To our knowledge, this is the first study to compare the effects of two short-term dietary interventions on CVC and cholesterol levels in a young, sedentary population. This research offers new information about the effectiveness of the MD and VD to impact several important health markers, and highlights that changes can be observed in as little as four weeks of intervention. This work adds to previous research from our lab [18], and data could now be beginning to suggest that short-term dietary modifications are sufficient to positively impact some cardiovascular risk factors.

### 4.1. Vegan Diet

Baseline data indicated that the groups’ habitual diets were comparable prior to intervention. Both treatments led to changes in dietary composition, and reductions in Saturated Fat and increases in Fibre were experienced by both groups. VD participants experienced a noteworthy reduction in TC, reflecting data elsewhere suggesting that TC levels are malleable to dietary modification [2,31]. Indeed, our findings echo those of McDougall et al. [32], who found that a seven-day low-fat VD intervention led to reductions in TC. In contrast to McDougall’s study however our participants were not instructed to follow a low-fat diet (10% of total Calories), and consumed a mean intake of 73.7 g/day (≈ 36.6% of total Calories) via ad libitum feeding, suggesting that the reduction in TC we observed might not just be the result of lowering Total Fat alone. The VD participants reduced Saturated Fat intakes from 38.2 g/day (16.3% of total Calories) to 14.6 g/day (7.2% of total Calories), which, when coupled with increases in soluble fibre (∆ fibre = 17.19 g ± 10.83), perhaps due to the increases in Vegetables, Fruits, and Legumes consumed (Table 3), might explain the reduction in TC we observed [2,28]. It should be noted that the increase in legume consumption, whilst a large increase (VD ∆ Legumes = 191.2 ± 148.3), did not reach statistical significance. Interestingly, these changes were not observed in the MD group, who did not adjust their Saturated Fat and Fibre intakes (nor increase fibre, grain, and legumes consumed) to the extent of the VD participants.

The health benefits of consuming adequate dietary fibre are beginning to be understood, specifically in the prevention and treatments of conditions such as type II diabetes, CVD and colon cancer [33,34]. In this study, the VD group increased their Fibre intake from 20.5 g/day to 37.7 g/day, and the MD group from 20.8 g/day to 26.9 g/day (∆ Fibre was notably greater in the VD group). Such increases could impact health, and future research could investigate long-term health effects of novel high-fibre diets, such as a VD or MD, for a variety of health markers sensitive to its intake, such as glycaemia and lipids [33,34]. Indeed, it is likely that the changes we observed in the VD group, such as reduced TC and weight loss, are at least partially explained by this shift in dietary composition [2,4,16,17,32,34].

Both groups ate less as a result of their diets (∆ Calories = −220 kcal/day for MD participants; −296 kcal/day for VD participants) despite being instructed to maintain energy consumption. The VD participants experienced weight loss (VD ∆ body mass = −1.0 kg; MD ∆ body mass = 0.17 kg), which was not an expected outcome of this research. While we cannot rule out that participating in a dietary intervention might prompt some participants to eat less, our findings appear to be consistent with research elsewhere highlighting weight loss associated with vegan diets [35]. Interestingly, this weight loss occurred following reductions in protein (VD ∆ Protein = −29.8 g/day) and fat (VD ∆ Total Fat = −23.47 g/day), and the increase in fibre (VD ∆ Fibre = 17.19 g/day). Data indicates that hypocaloric protein-rich diets assist weight loss due to their satiating properties [36]. Similar benefits have also been reported for high-fibre diets [37]. The VD group experienced their weight loss despite consuming less protein; increases in fibre therefore might have offset any possible reductions in satiety following the reduction in protein [37], and facilitated this weight loss. Whilst we did not measure satiety specifically, future research could investigate the satiety effects of a hypocaloric vegan diet, and research into possible weight-loss effects of vegan diets with various macronutrient (high vs. low protein, etc.) and fibre compositions in comparison to other dietary approaches.

It should be noted, however, that the VD group experienced marked reductions in a number of important micronutrients, including Iodine (∆ Iodine = −150.2) and Vitamin B12 (∆ Vitamin B12 = −4.0) by following their intervention. Such sizeable reductions should be of concern to individuals who adopt a vegan diet, as poor micronutrient status can have severe health implications over the long term. We provided participants with a Vitamin B12 supplement to offset an expected reduction in Vitamin B12 intake, however future studies might need also to be cognisant of the potential for other micronutrients, such as Sodium, Chloride, Phosphorous, Iodine, and Riboflavin to be reduced significantly too; and to offer suitable strategies to recompense for a reduced intake, if warranted.

### 4.2. Mediterranean Diet

Measuring microvascular reactive function via LDF facilitates the evaluation of endothelial health [21]. The endothelium is a selectively-permeable vascular interface between blood and tissue [21]. Endothelial dysfunction is characterised by diminished vasodilation and reduced nitric oxide supply, and is a precursor to CVD [3]. Emerging data highlights that lifestyle modifications can improve endothelial function [7,18,19,20]. Our findings add to this evidence, and similar to Klonizakis et al. [19] and Alkhatib and Klonizakis [20], we observed improvements in microvascular function in response to following the MD. In our study, baseline CVC was similar for both groups. However, following the four-week intervention, MD participants experienced improvements in plateau raw CVC that were not experienced by the VD group. This improvement in plateau CVC suggests that endothelial-mediated nitric oxide (NO) availability improved in response to the MD [38]. Impaired NO availability is characteristic of endothelial dysfunction, meaning that strategies that maintain or improve endothelial-NO levels are important in mitigating CVD risk [21,38,39]. Plant-based foods such as leafy green vegetables and beetroot are rich in dietary (inorganic) nitrates, and evidence demonstrates that dietary nitrates might increase NO levels via the nitrate-nitrate-nitric oxide pathway [39]. It is interesting therefore that the VD participants did not experience similar changes considering that the VD is wholly plant-based. However, it should be noted that we did not instruct participants to consume nitrate-rich foods specifically in this research, nor did we equate vegetable and fruit consumption between the groups, meaning that the differences in CVC data could be due to the MD group favouring the consumption of nitrate-rich vegetables. However, our findings confirm existing data elucidating the endothelial-health-promoting benefits of the MD [7,19,20], making this effect unlikely, and suggesting that the improvements observed in the MD group might be attributable to other dietary factors.

The MD promotes the consumption of Olive Oil, which possesses multiple health benefits due to its rich polyphenol content [40]. Indeed, participants who completed the MD intervention in this study increased their consumption of Olive Oil notably (MD ∆ Olive Oil = 17.7 ± 9.9), as it appeared that participants did not consume Olive Oil as part of their habitual diets prior to the commencement of this study. Interestingly, this increase in consumption was coupled with a concurrent decrease in Other Oils and Fats (MD ∆ Other Oils and Fats = −13.4 ± 20.4), suggesting that participants swapped their consumption of Other Oils and Fats for Olive Oil specifically. Extra virgin olive oil (in particular) has been shown to possess strong antioxidant properties due to its abundant polyphenol content (particularly oleuropein), and causal links are known to exist between oxidative stress, inflammation, endothelial dysfunction, and CVD [39]. Olive oil is also rich in Oleic acid (an omega-9-monounsaturated fatty acid), which might reduce inflammation and improve metabolic and lipid health similarly [7,40]. It is possible therefore that such bioactive compounds augment the microvascular benefits of following the MD, and that the health effects of the diet are the result of a complex interplay between food elements. Indeed, making inferences about its mechanism of action are challenging given the complexity of its nutritive composition. Nevertheless, more research is necessary to investigate and isolate the health-promoting components of the MD, and other novel dietary strategies similarly.

Limitations of this research were a lack of randomisation, a small sample size with unequal numbers of male and female participants, and the short intervention period. However, in pilot studies that employ parallel-groups designs such as this research, sample sizes of 12 per group are considered to be appropriate, based on factors such as feasibility and precision around estimates to design further work [41]. Indeed, the purpose of this research is to inform further research, and our data features novel findings that should now inform larger, longer-term randomised controlled trials. Our approach to recruitment was pragmatic, and robust measures were taken to account for all known factors that affect microcirculation. We also tested female participants on specific days to minimise gender effects of sex hormone cycles. Therefore, whilst randomisation did not take place, we are confident that for the main area of interest in this research (microcirculation) the groups were balanced. Future research, however, might wish to isolate and investigate the effects of novel diets on the different genders specifically. It should also be noted that the intervention period of this study was intentionally short, to corroborate if meaningful improvements in microcirculatory health markers could be observed in as little as four weeks of intervention, reflecting data previously reported by our lab [42]. Elucidating these findings might help to define the prescription of specific diets in different populations, and our findings add to growing evidence in this area. The microcirculation effects we observed occurred within normal parameters of a young and healthy, sedentary population; however, these findings add to existing data from our lab, which indicate that short-term dietary interventions can elicit improvements in other populations similarly, and outside of normal parameters [19,20,42]. Therefore, based upon our preliminary findings, this study could now help to inform the design and development of future, larger dietary intervention studies, and should serve as impetus for additional research in the areas discussed herein. 

## 5. Conclusions

This study highlighted the possible short-term health effects of following a four-week VD and MD intervention, revealing that the VD led to reduced TC and weight loss, and that the MD led to improvements in microvascular function (in general) and NO bioavailability (specifically) in a healthy sedentary population. While both diets promoted a micronutrient-rich diet, the health effects of the diets differed, suggesting that the nuances of each might offer different health outcomes when compared empirically. More research is necessary to elucidate and understand the possible health effects of such novel diets.

## Figures and Tables

**Table 1:** Dietary Interventions: Mediterranean Diet vs. Vegan Diet.

Mediterranean Diet ^a^	Vegan Diet ^b^
Extra virgin olive oil (≈4 Tbsp/day) Vegetables (2–3 servings/day) Fruit (2–3 servings/day) Tubers White meat (instead of red meat) Legumes (3 servings servings/week) Low fat dairy Products (2–3 servings/day) Red meat (discouraged) Wine with meals (optional)	Vitamin B12 supplement (2.6 μg/day) Fruit and Vegetables (>5 servings/day) Protein foods (beans, peas, lentils, soya products) (3 + servings/day) Nuts, seeds (2–3 servings/day) Whole grains (each meal) Calcium-fortified Plant milks and dairy alternatives (2–3 servings/day) No animal products

a: Adapted from Estruch et al. [26]; b: Developed from Phillips [27]. Serving sizes based on weight; 75 g (100–350 KJ) for vegetables; 150 g (350 KJ) fruit; 75–120 g (500 KJ) for cereals (cooked); 500–600 KJ for meats (≈100 g raw), legumes (150 g cooked), nuts and seeds (30 g); 250 mL (500–600 KJ) for dairy and/or alternatives.

**Table 2:** Participants’ Characteristics.

	Mediterranean Diet			Vegan Diet		
	Visit 1	Visit 2	Difference	Visit 1	Visit 2	Difference
Gender	2 male 10 female			4 male 8 female		
Age (years)	25 (2.6)			26 (4.3)		
Stature (m)	1.7 (0.07)			1.71 (0.07)		
Body Mass (kg)	66.9 (12.8)	67.0 (13.0)	0.17 (1.06) **	73.2 (18.6)	72.1 (17.6) *	−1.05 (1.53) **
BMI (kg∙m^2^)	23.1 (3.2)	23.2 (3.3)	0.06 (0.36) **	25.1 (6.5)	24.7 (6.1)	−0.36 (0.54) **
Systolic BP (mmHg) ^†^	107.9 (8.8)	108.8 (7.8)	0.94 (7.58)	119 (9.5)	117.6 (13.0)	−1.45 (10.09)
Diastolic BP (mmHg) ^†^	68.2 (7.3)	68.1 (6.3)	−0.08 (8.14)	76.4 (11.0)	77.1 (13.0)	0.73 (7.48)
MAP (mmHg)	85.1 (47.3)	90.6 (8.5)	5.50 (10.95)	90.7 (9.3)	90.6 (12.4)	−0.09 (11.70)
TC (mmol/L) ^†^	4.20 (0.67)	4.15 (0.75)	−0.05 (0.74)	3.88 (0.56)	3.52 (0.56) *	−0.36 (0.52)
HDL-C	1.50 (0.61)	1.49 (0.45)	−0.01 (0.35)	1.17 (0.49)	1.16 (0.34)	−0.01 (0.26)
TC:HDL-C	3.41 (2.00)	2.99 (0.92)	−0.42 (1.31)	3.85 (1.60)	3.30 (1.18)	−0.55 (0.89)

* *p* ≤ 0.05 between visits (within groups); ^**^ *p* ≤ 0.05 between groups; ^†^ *p* ≤ 0.05 between groups (at both time points).

**Table 3:** Food Group Data.

	Mediterranean Diet			Vegan Diet		
	Visit 1	Visit 2	Difference	Visit 1	Visit 2	Difference
Dairy products (g) ^a^	150 (180.4)	165.1 (158)	15.1 (136.6)	373.1 (403.2)	313 (314.9)	−60.1 (239.6)
Meat and Poultry (g)	181.1 (98.8)	132.7 (176.8) ^†^	−48.4 (189.5)	173.3 (264.4) *	0 (0) ^†,^*	−173.3 (264.4)
Fish	56.3 (64.2)	41.9 (36.9) ^†^	−14.4 (81.5)	33.8 (60.91)	0 (0) ^†^	−33.8 (60.9)
Eggs	62.1 (53.9)	36.7 (69.9)	−25.4 (92.9)	57.5 (28.4) *	0 (0) *	−57.5 (28.4)
Vegetables	165.2 (142.5) *	263.3 (83.6) *	98.1 (100.9)	132.1 (11.6) *	279.1 (165) *	147.1 (113.6)
Fruit	17.6 (59.7)	187.8 (123.9) ^†^	170.2 (143.5) ^†^	121.4 (114.1) *	401.5 (352.5) ^†,^*	280.1 (357.9) ^†^
Tubers	57.8 (48.5)	39.7 (102.3)	−18.1 (113.1)	90 (142.5)	50 (60)	−40 (114.6)
Cereals	264.0 (152.9)	294.9 (101.2)	30.9 (96.6)	199.2 (102)	285.8 (125.7)	86.6 (141.2)
Refined Cereals	32.9 (40.4)	11.7 (12.5)	−21.3 (37.7)	36 (68) *	15 (18.9) *	−21 (73.6)
Legumes	34.4 (84.4)	85.4 (110)	51 (120.2)	21 (59.6)	212.2 (133.6)	191.2 (148.3)
Nuts and Seeds	0 (0)	7.7 (7.8) ^†^	7.7 (7.8)	9.8 (19.8) *	59.6 (75.2) *^,†^	49.8 (80.9)
Olive Oil	0 (0) ^†,^*	14.7 (6.9) *	14.7 (6.9) ^†^	7 (6.3) ^†,^*	8.2 (6.2) *	1.2 (10.5) ^†^
Other Oils and Fats ^b^	16.5 (16.2) *	3.1 (6.9) *	−13.4 (20.4) ^†^	9.6 (7.7)	5.3 (5.7)	−4.28 (9.7) ^†^
Sweets ^c^	39.8 (22.8)	1.3 (0)	−38.4 (22.8)	86 (181.9)	12.6 (0)	−73.3 (188.4)
Alcohol	5.3 (9.2) *	1.9 (5.7) ^†,^*	3.4 (9.1) ^†^	0 (0)	0.1 (0.3) ^†^	0.1 (0) ^†^

All values are mean g/day ± SD; * *p* ≤ 0.05 between visits (within groups); ^†^ *p* ≤ 0.05 between groups; ^a^ includes dairy replacement products; ^b^ includes all oil and fat sources not from olive oil; ^c^ includes sugars sweetened beverages.

**Table 4:** Macronutrient Data.

	Mediterranean Diet			Vegan Diet		
	Visit 1	Visit 2	Difference	Visit 1	Visit 2	Difference
Calories (kcal)	2087.9 (589.9)	1867.9 (516.7)	−220.00 (476.21)	2108.3 (757.3)	1812.0 (541.5)	−296.3 (736.49)
CHO (g)	220.9 (53.8)	199.0 (53.3)	−21.92 (66.27)	221.1 (78.3)	230.2 (89.0)	9.10 (98.68)
Protein (g)	83.0 (36.5)	82.3 (31.3) ^†^	−0.66 (26.46)	86.9 (41.4)	57.2 (18.6) *^,†^	−27.77 (38.93)
Total Fat (g)	88.2 (32.8)	73.8 (28.8) *	−14.33 (21.91)	97.2 (40.2)	73.7 (23.4) *	−23.47 (34.93)
Mono. Fat (g)	30.3 (12.4)	32 (11.5)	1.7 (14.7)	31.2 (9.8)	29.8 (9.7)	−1.42 (10.6)
Poly. Fat(g)	13.4 (5.4)	14.9 (6.4)	1.5 (5.6)	13.5 (6.4)	17.9 (8.3)	4.39 (6.7)
Omega 3 (g)	1.4 (1.1)	1.4 (1.1)	0 (0.7)	0.8 (0.5)	1.5 (1.2)	0.7 (1.1)
Omega 6 (g)	7.4 (3.4)	6.8 (3.1)	−0.6 (3.5)	6.5 (4.4)	13.5 (6.9)	7.0 (6.7)
Sat. Fat (g)	30.7 (13.1)	20.0 (11.7) **^,†^	−10.72 (6.39)	38.2 (22.1)	14.5 (5.6) *	−23.62 (19.45) ^†^
Fibre (g)	20.1 (5.8)	26.9 (6.7) *^,†^	6.17 (5.27) ^†^	20.5 (6.3)	37.7 (11.9) **^,†^	17.19 (10.83) ^†^

All values are mean g/day ± SD; * *p* ≤ 0.05 between visits (within groups); ** *p* ≤ 0.001 between visits (within groups); ^†^ *p* ≤ 0.05 between groups.

**Table 5:** Micronutrient Data.

	Mediterranean Diet			Vegan Diet		
	Visit 1	Visit 2	Difference	Visit 1	Visit 2	Difference
Sodium (mg)	1569 (763)	2146 (785) ^†^	577 (914) ^†^	2092 (1080) *	1161 (852) *^,†^	−931 (586) ^†^
Potassium (mg)	3151 (722)	3142 (804)	−9 (418)	2796 (1136)	3063 (687)	267 (1378)
Chloride (mg)	2693 (1092) *	3612 (1343) *^,†^	919 (713) ^†^	2967 (1394) *	1512 (478) *^,†^	−1455 (1267) ^†^
Calcium (mg)	747 (271)	836 (235)	89 (211) ^†^	988 (593)	669 (593)	−318 (455) ^†^
Phosphorus (mg)	1337 (490)	129 (472)	−45 (248) ^†^	1367.6 (491) *	1020 (269) *	−348 (449) ^†^
Magnesium (mg)	423 (135) ^†^	412 (101)	−11 (99) ^†^	283.9 (104) *^,†^	375 (139) *	91 (98) ^†^
Iron (mg)	12.1 (3) *	13.6 (3) *	1.5 (2) ^†^	9.7 (3.4) *	13.5 (5) *	3.8 (3) ^†^
Zinc (mg)	8.5 (2)	9.3 (3) ^†^	0.8 (2) ^†^	9.2 (3.7)	7.0 (2.4) ^†^	−2.2 (3.5) ^†^
Copper (mg)	1.3 (0)	1.6 (0)	0.3 (0) ^†^	1.1 (0.4) *	1.9 (0.8) *	0.8 (0.5) ^†^
Manganese (mg)	3.2 (1) *	3.8 (1) *^,†^	0.6 (1)	3.6 (1.4) *	5.8 (1.9) *^,^^†^	2.2 (1.9)
Selenium (µg)	52.6 (28)	65.7^†^ (34)	13.1 (18)	50.2 (31.4)	38.1^†^ (18.4)	−12 (36.8)
Iodine (µg)	145 (86)	131 (49) ^†^	−14 (72)	169.3 (190.6) *	19.05 (6.9) *^,†^	−150 (189.7)
Vitamin A (µg)	627 (438) *	1054 (562) *	427 (417)	799 (553)	1123 (1078)	324 (1189)
Vitamin D (µg)	3.4 (2)	4.3 (3)	0.9 (2)	2.4 (2)	2 (2)	−0.4 (5.6)
Vitamin E (mg)	10.1 (4)	10.7 (4)	0.6 (2)	9.7 (5.6)	11.6 (6.9)	1.9 (6)
Vitamin K_1_ (µg)	78.7 (94)	113.7 (77)	34.9 (52)	75.15 (138)	74.7 (62)	−0.45 (99.7)
Thiamin (mg)	1.5 (0) *	2.2 (1) *	0.7 (1)	1.5 (0.6)	1.6 (0.6)	0.1 (0.8)
Riboflavin (mg)	1.6 (1)	1.7 (1) ^†^	0.1 (1)	1.7 (0.9) *	1.1 (0.5) *^,†^	−0.58 (0.6)
Niacin (mg)	40.2 (7)	47.9 (28)	7.7 (12)	32 (11)	23 (11)	−9 (15)
Pantothenic Acid (mg)	5.6 (3)	6.3 (3) ^†^	0.7 (1)	5.5 (3)	3.5 (1) ^†^	−2 (3.3)
Vitamin B6 (mg)	1.8 (1)	2.3 (1) ^†^	0.5 (1)	1.7 (0.8)	1.4 (0.4) ^†^	−0.2 (1)
Folic Acid (µg)	277.9 (91)	294.8 (84)	16.9 (70)	223.4 (108)	272.2 (95)	48.8 (87)
Vitamin B12 (µg) **	4.6 (2)	5 (2) ^†^	0.4 (2)	4.9 (3) *	0.9 (1) *^,†^	−4 (2.5)
Biotin (µg)	34(14) *	36 (6) *	2 (12)	34 (15)	41 (23)	7 (26)
Vitamin C (mg)	97.7(50)	116 (42)	18 (49)	107.4 (83)	109.3 (72)	1.9 (96)

All values are mean mg/day ± SD unless stated; * *p* ≤ 0.05 between visits (within groups); ^†^ *p* ≤ 0.05, between groups; ** values excluding B12 supplement (Vegan Diet group only).

**Table 6:** CVC Data.

	Mediterranean Diet		Vegan Diet	
	Raw CVC	%CVC MAX	Raw CVC	%CVC MAX
**Baseline**				
Visit 1 (pre-intervention)	0.19 (0.1)	6.64 (3.65)	0.2 (0.08)	8.8 (6.2)
Visit 2 (post-intervention)	0.24 (0.1)	6.03 (4.25)	0.2 (0.1)	6.7 (3.3)
Difference	0.05 (0.15)	−0.65 (5.66)	−0.03 (0.08)	−2.13 (6.78)
**Initial Peak**				
Visit 1 (pre-intervention)	2.24 (0.6)	77.9 (20.4)	1.96 (0.8)	66.4 (13.5)
Visit 2 (post-intervention)	3.14 (0.8) *	74.6 (16.0)	2.05(1.3)	63.5 (13.9)
Difference	0.90 (0.8) ^†^	−3.29 (11.3)	0.09 (0.9) ^†^	−2.88 (21.7)
**Plateau**				
Visit 1 (pre-intervention)	2.59 (0.7)	89.5 (21.1)	2.55 (1.0)	84.6 (7.2)
Visit 2 (post-intervention)	3.32 (0.8) *	78.4 (11.7)	2.71 (1.5)	83.3 (6.0)
Difference	0.73 (0.95)	−11.1 (30.1)	0.17 (0.83)	−1.37 (10.1)

^†^ *p* ≤ 0.05 between groups; * *p* ≤ 0.05 between visits (within groups).
